# SARS-CoV-2 Serology Monitoring of a Cancer Center Staff in the Pandemic Most Infected Italian Region

**DOI:** 10.3390/cancers13051035

**Published:** 2021-03-02

**Authors:** Chiara Maura Ciniselli, Arianna Micali, Loris De Cecco, Paola Notti, Valentina Sinno, Elena Luison, Cecilia C. Melani, Maria Grazia Daidone, Giovanni Apolone, Paolo Verderio, Mariangela Figini

**Affiliations:** 1 Unit of Bioinformatics and Biostatistics, Department of Applied Research and Technological Development, Fondazione IRCCS Istituto Nazionale dei Tumori (INT), 20133 Milan, Italy; chiara.ciniselli@istitutotumori.mi.it; 2 Integrated Biology Platform, Department of Applied Research and Technology Development, Fondazione IRCCS Istituto Nazionale dei Tumori (INT), 20133 Milan, Italy; arianna.micali@istitutotumori.mi.it (A.M.); loris.dececco@istitutotumori.mi.it (L.D.C.); 3 Medical Directorate, Fondazione IRCCS Istituto Nazionale dei Tumori (INT), 20133 Milan, Italy; paola.notti@istitutotumori.mi.it; 4 Clinical Trials Center, Fondazione IRCCS Istituto Nazionale dei Tumori (INT), 20133 Milan, Italy; valentina.sinno@istitutotumori.mi.it; 5 Biomarker Unit, Department of Applied Research and Technology Development, Fondazione IRCCS Istituto Nazionale dei Tumori (INT), 20133 Milan, Italy; elena.luison@istitutotumori.mi.it (E.L.); mariagrazia.daidone@istitutotumori.mi.it (M.G.D.); mariangela.figini@istitutotumori.mi.it (M.F.); 6 Scientific Directorate, Fondazione IRCCS Istituto Nazionale dei Tumori (INT), 20133 Milan, Italy; cecilia.melani@istitutotumori.mi.it (C.C.M.); giovanni.apolone@istitutotumori.mi.it (G.A.)

**Keywords:** COVID-19, serology, healthcare personnel, comprehensive cancer center, antibody response

## Abstract

**Simple Summary:**

Since the beginning of the COVID-19 outbreak, Cancer Centers adopted specific procedures to protect patients as well as to monitor the possible spread of SARS-CoV-2 among healthcare personnel. In April 2020, we implemented a prospective longitudinal study aimed at monitoring the serological response to SARS-Cov-2 in the healthcare personnel of a Comprehensive Cancer Center identified by the Lombardy region (the Italian region most affected by the COVID-19 pandemic) as one of the three oncologic hubs where the Regional Health Authorities referred all the cancer patients in the region. We identified a fraction of healthcare personnel with long-term anti-SARS-CoV-2 antibody response, though negative for viral RNA, who could safely approach fragile cancer patients.

**Abstract:**

Since the beginning of the COVID-19 outbreak, Cancer Centers adopted specific procedures both to protect patients and to monitor the possible spread of SARS-CoV-2 among healthcare personnel (HCP). In April 2020 at Fondazione IRCCS Istituto Nazionale dei Tumori, Milano, one of the three oncologic hubs in Lombardy where the Health Regional Authorities referred all the cancer patients of the region, we implemented a prospective longitudinal study aimed at monitoring the serological response to SARS-Cov-2 in HCP. One hundred and ten HCP answered a questionnaire and were screened by nasopharyngeal swabs as well as for IgM/IgG levels; seropositive HCPs were further screened every 40–45 days using SARS-CoV-2-specific serology. We identified a fraction of HCP with long-term anti-SARS-CoV-2 antibody responses, though negative for viral RNA, and thus probably able to safely approach fragile cancer patients. Monitoring asymptomatic HCP might provide useful information to organize the healthcare service in a Cancer Center, while waiting for the effectiveness of the active immunization by SARS-CoV-2 vaccines, which will provide protection from infection.

## 1. Introduction

Several recently published papers [1,2] reported contrasting results about the longevity of antibody titers to SARS-CoV-2 and, in general, there is no full agreement in literature on this issue. Although results from some authors seem to support rapid waning of virus-specific IgG antibodies up to approximately three months after infection [3,4], others detected substantial stable titer levels detected over several weeks or several months [5,6,7,8].

Since the beginning of the COVID-19 outbreak, Cancer Centers have adopted specific procedures to protect patients, as well as to monitor the possible spread of SARS-CoV-2 among healthcare personnel (HCP) [9,10]. At the Fondazione IRCCS Istituto Nazionale Tumori (INT) of Milan, a Comprehensive Cancer Center that became one of the three hubs for oncologic patient referral in Lombardy (the Italian region most affected by the COVID-19 pandemic) we implemented in April 2020 a prospective longitudinal study aimed at: (a) screening HCPs without overt symptoms by nasopharyngeal swabs (NPS) and (b) monitoring their IgM/IgG levels every 40–45 days by SARS-CoV-2-specific serology to assess the persistence of humoral immunity, in combination with the possible occurrence of COVID-19 symptoms as recorded by a questionnaire.

Monitoring HCP without overt symptoms during the first and second wave of pandemic in Lombardy proved useful to organize the healthcare service in our Cancer Center and will be helpful to test the effectiveness of the SARS-CoV-2 vaccination.

## 2. Materials and Methods

HCP were prospectively recruited in order to obtain a representative sample of the different professional categories at the INT. All participants signed an informed consent, and the study was reviewed and approved by the Internal Review Board and Ethics Committee. 

Between 9th and 29th April, 110 HCP answered a questionnaire and were sampled by venipuncture and NPS. HCP seropositive at admission (T_0_) underwent three additional blood samplings on 11th June (T_1_), 22nd–23rd July (T_2_) and 7–9th September (T_3_). 

Viral RNA was extracted from nasopharyngeal swabs (Virus RNA Collection kit, LCM-Genect, Italy) using DSP Virus/Pathogen midi kit automated on QIAsymphony instrument (Qiagen, Germany). SARS-CoV-2 positivity was investigated in all cases at T_0_ by real-time polymerase chain reaction (RT-PCR) for N, ORF1ab and E mRNA. In detail, ORF1ab and N genes were detected using the VIASURE SARS-CoV-2 RT-qPCR (CerTest-Biotec, Spain) following the manufacturer’s instructions. The Gene E probe set was synthesized by Integrated DNA Technologies (IDT, TEMAricerca, Italy) based on Corman et al. [11] and detected following the Centre for Disease Control and Prevention (CDC, Atlanta, GA, USA) protocol. Samples were run in duplicate and the data were collected by QuantStudio 12 K Flex software v1.2.3 (ThermoFisher, Carlsbad, CA, USA). Positivity for the presence of viral RNA in the clinical specimens was established when at least two genes were detected at Ct < 38. 

Plasma samples were challenged for anti-SARS-CoV-2 IgM/IgG by Enzyme-Linked Immunosorbent Assay (ELISA) supplemented by a confirmatory test for IgG specificity to the immunodominant antigens, Nucleocapsid (NC), and Spike protein, S1 and S2 subunits (Diagnostic Bioprobes, Dia.Pro, Sesto San Giovanni, Milan, Italy) [12] according to the manufacturer’s recommendations. The assay was developed with 3,3′,5,5′-tetramethylbenzidine (TMB) and readings at 450 nm/620–630 nm after blocking with sulfuric acid. One blank, two negative, and one positive control were included in each test. Results (S/Co) were calculated by dividing the Optical Density (OD) value of the sample by the cut-off value determined with the following formula, based on the mean of the negative controls (N_C_): Cut-off (Co) = N_C_ + 0.25. A lateral flow immunochromatographic test (PRIMA LAB SA, Switzerland) and the ChemiLuminescence ImmunoAssay (CLIA) DiaSorin test (DiaSorin, Saluggia, Italy; FDA-EUA) were also applied on plasma samples at T_0_. 

All data were recorded in a dedicated electronic-Case Report Form (e-CRF). Subject characteristics of the cohort were summarized using basic descriptive statistics. Assay data were firstly interpreted qualitatively according to the manufacturer’s recommendations, then the time trends profiles of the seropositive HCP at T_0_ were assessed by resorting to mixed models by considering, as the pivotal variable, the Dia.Pro S/Co values (on logarithmic scale) as a function of time [13]. In these models, the most appropriate matrix of variance-covariance was selected according to the Akaike Information Criterion (AIC). Statistical analyses were carried out with SAS software (Version 9.4; SAS Institute, Inc., Cary, NC, USA) and graphical representations were performed with R-software (ggplot2 package). 

## 3. Results

The 110 HCP ranged from 23 to 69 years old (median: 49 yrs) and 55% were female; physicians represented 40% of the study sample, nurses 29%, health workers 25%, administrative personnel 2% and laboratory personnel or other 4%. A close contact with a confirmed or putative COVID-19-affected case was notified by 54% of HCP (14% outside INT and 83% within INT’s premises). Among the 110 HCP 60% reported no symptoms, 24% only 1–2 mild symptoms and the remaining 16% declared at least three symptoms (headache 72%, fatigue/malaise 66%, myalgia 61%, arthralgia 56%, sore throat 50%, rhinorrhea 38%).

All but two subjects were negative for the RT-PCR analysis; the two suspicious cases revealed Ct ≤ 37 for the N gene, Ct ≤ 34 or < 38 for the E gene and absence of detection for ORF1ab. At T_0_, 21/110 HCP had anti-SARS-CoV-2 IgG (19%) and seven had IgM (6%) antibodies, six of whom were also IgG positive according to the Dia.Pro ELISA assay. Among IgG-positive subjects, the confirmation ELISA detected anti-NC in 19 cases out of 21, and anti-S1 and anti-S2 in eight and three cases respectively: IgG specificity for all of the three antigens was detected only in 3/21 HCPs. The two cases with uncertain RT-PCR values proved to be positive for IgG to NC and S1 at T_0_. The subjects positive for NC and spike were also found positive with PRIMA and DiaSorin (Table 1). 

Twenty-two out of the 110 HCP (20%) resulted seropositive at T_0_ according to both IgG and IgM Dia.Pro tests. Nineteen accepted to continue the longitudinal study. Only three of them reported at least three symptoms: all reported headache, two also reported fatigue/malaise, myalgia and arthralgia (one also with conjunctivitis) and one augeusia/disgeusia. 

**Figure 1:**
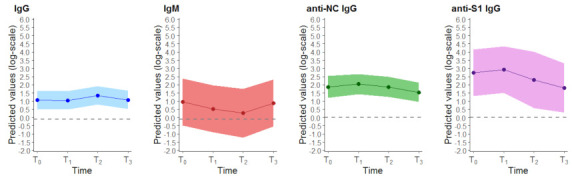
Time trend profiles for the presence of antibodies to SARS-CoV-2 in healthcare personnel with a positive T_0_ result. Colored dot points indicate the predicted mean value at each time, and the solid lines the estimated time trend together with the 95% confidence band. The grey dashed line indicates the assay cut-offs for positivity.

Figure 1 depicts the S/Co time trend profiles of IgG, IgM, anti-NC and anti-S1IgG, respectively, by considering only subjects with a positive T_0_ result (*n* = 18, 6, 18 and 7 for IgG, IgM, anti-NC and anti-S1 IgG, respectively).

## 4. Discussion

In consideration of recurrent COVID-19 pandemic waves and waiting for the effectiveness of anti-SARS-CoV-2 vaccines, our sero-survey was built to monitor putative humoral immunity, not in subjects with overt symptoms but in presumed COVID-19 free HCP unable to comply with the lockdown regulations due to their work duties, and who were in contact with fragile and immunosuppressed oncologic patients more prone to become SARS-CoV-2 targets. 

In a relevant proportion of mostly asymptomatic NPS-negative HCP we detected an anti-SARS-CoV-2 seropositivity which persisted after six months with a modest decrease in anti-NC and anti-S1 IgG. This suggests their probable contact with the virus in the past, or at the very beginning of the study, but without overt symptoms in the study time frame. Besides the stability of anti-NC antibodies, it is worth noting that IgG persists to Spike-1, which is proved to correlate with neutralizing antibodies [14]. 

It is likely that seropositive subjects experienced a mild COVID-19 presentation, since they were enrolled during the peak of the first epidemic wave in Lombardy, the most affected region in Italy. Our sero-surveys, now extended to more HCP, lacked functional assays, although T_0_ results were confirmed by alternative CE-certified tests, so we cannot infer on the protective activity of these IgG. 

To grasp as much information from the data as possible, we also evaluated the S/Co values in the HCP seropositive at T_0_ over time by depicting the longitudinal profile of the considered antibodies. In disagreement with some reports [4,7], but in agreement with others [8] on asymptomatic individuals, the length of the immune response in our HCP could be longer than 150 days. We intend to continue our sero-surveys over time to investigate possible protection from reinfection during the new pandemic waves and to provide useful information for the efficacy of SARS-CoV-2 vaccines.

## 5. Conclusions

Monitoring asymptomatic HCP might provide information useful to the organization of the healthcare service in a Cancer Center while waiting for the active immunization by SARS-CoV-2 vaccines, which will provide protection from infection.

## Figures and Tables

**Table 1:** Performance at T_0_ of the Dia.Pro immunoassay (total IgG and confirmation test) compared to alternative approaches.

Dia.Pro Immunoreactivity by ELISA (Number of Cases)				
	Total IgG ^a^		Confirmation Test ^b^ (on 21 IgG-Positive Cases)	
	Negative (*n* = 89)	Positive (*n* = 21)	NC (*n* = 19)	S1/S2 (*n* = 8)
**PRIMA Test**				
Negative	88	14 (0)	12	1
Positive	1	7 (2)	7	7
**DiaSorin Test ^c^**				
Negative	88	16 (0)	14	3
Positive	1	5 (2)	5	5

^a^ Dia.Pro cut-off for positivity: ≥0.9 S/Co; ^b^ Dia.Pro confirmation test cut-off for positivity: ≥1 S/Co; ^c^ DiaSorin cut-off for positivity: ≥12 units; in brackets the number of suspicious cases according to real-time polymerase chain reaction (RT-PCR).

## Data Availability

The data presented in this study are available on request from the corresponding author.
